# A method for comparing perceptual distances and areas with multidimensional scaling

**DOI:** 10.1016/j.mex.2020.100790

**Published:** 2020-01-18

**Authors:** Phil J. Howson, Philip J. Monahan

**Affiliations:** a University of Oregon, Department of Linguistics, United States; b University of Toronto, Department of Linguistics, Canada; c University of Toronto Scarborough, Centre for French & Linguistics, Canada; d University of Toronto Scarborough, Department of Psychology, Canada

**Keywords:** Distance and area comparison with multidimensional scaling, Speech perception, Linguistics, Principle Coordinate Analysis

## Abstract

This paper presents a method for adding additional statistical comparisons to multidimensional scaling (MDS). The object of study in our work is perceptual distances between speech sound categories. Typically, MDS solutions do not receive inferential statistical treatment and their visualizations present average results across numerous participants. This is problematic because it ignores inter-participant variation. To account for this variance, we have devised a simple technique for adding statistical power to the traditional MDS solution so that the distances between objects and the areas occupied by groups of objects can be compared more reliably than visual inspection of an MDS plot. We provide a method for comparing distances between two objects and for comparing the area of three or more objects. This method can be paired with varying statistical analysis to suit the researcher's needs.

• Adds statistical power to multidimensional scaling.
• Compares distances between segments.
• Compares dispersion of groups of objects in multidimensional space.

## Graphical abstract

**Figure:**
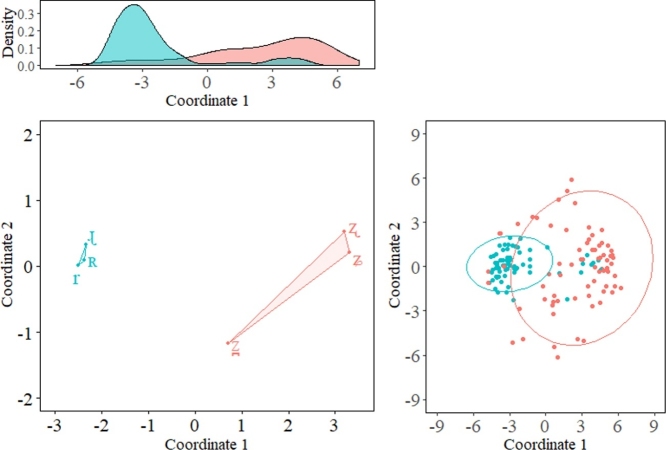

**Specification Table**

**Table:** 

Subject Area:	Social Sciences
More specific subject area:	Speech perception, Linguistics
Method name:	Distance and area comparison with multidimensional scaling
Name and reference of original method:	Multidimensional Scaling.
	Gower, J. C. & Legendre, P. (1986). Metric and Euclidean properties of dissimilarity coefficients. Journal of Classification 3, 5–48.

## Method details

### Background on multidimensional scaling

Principle Coordinate Analysis (PCoA; [1,2]), better known as Multidimensional scaling (MDS; [3]), is a method for visualizing the degree of dissimilarity between groups of objects. MDS is a commonly used measurement in psychophysics and sensory analysis. MDS calculates a series of Euclidean distances based on an input set of dissimilarity coefficients. The MDS model minimizes the *loss function* for a best fit model of the distances between the input objects. The configuration of *n* points within an abstract space is next mapped based on the pairwise distances among the input *n* objects [4]. Data is then plotted on an abstract and typically two-dimensional Cartesian space to provide visual information about the differences between objects; however, in many disciplines, such as Linguistics or Psychology, the input matrix is often the grand observation variable means of many participants and ignores individual variation. As a result, it is hard to ascertain how statistically dissimilar two or more objects are. Mead [4] describes this as a problem because when we discount the actual responses of each individual in a group, we cannot be sure that the average response matrix accurately represents consistent responses of each individual. Mead [4] also notes that simply analyzing each participants’ scores individually and describing recurring patterns is problematic because it requires a large dataset for each participant. Our method provides additional statistical power for MDS. It permits calculations of distances between objects or the amount of space that a group of objects occupies within the Cartesian space while taking into account variance amongst the individual participants. In this paper, we describe how to calculate the MDS coordinates and compare both distances and areas of the MDS solution. This method allows for visualization of the data that provides estimates of inter-participant variation and provides for greater statistical power, permitting the application of inferential statistical tests.

There are many ways to calculate an input dissimilarity matrix for use in an MDS solution. In this article, we used d-prime, a measurement of discrimination sensitivity [5], as the underlying observation variable. However, in linguistics, it is also common to use confusion data [6,7] and reaction time measures [8,9] to generate the dissimilarity matrix. We discuss our current method with respect to a subset of the d-prime data in our perceptual experiment [10]. We provide detailed information on how we performed our calculations with that experiment as an example; however, any dataset from which the creation of a dissimilarity matrix is possible can be used with our methods.

### Calculating the multidimensional scaling coordinates

We elaborate on this method here based on data from the fricatives condition of our perceptual experiment [6]. We performed an AX discrimination task [11] with 25 participants. The task included three rhotic segments, /r ɻ ʀ/, and three fricative segments, /z̪ ʐ ʑ/. Each participant heard 6 repetitions of each of the possible comparisons for each segment. This produced a total of 210 trials (60 same comparisons and 150 different comparisons), but we balanced the design for an equal number of same and different responses, so we included 90 more same comparisons (15 of each same comparison). Therefore, we had a total of 300 trials per participant (total 7500 trials). Responses faster than 300 ms and 2300 ms were discarded [12]. We then calculated the d-prime measures for each participant.

Once all the d-prime measures were calculated, we performed a classical MDS solution in R [13] using the cmdscale() function for each participant. Crucially, an input dissimilarity matrix must be calculated for each participant and the output coordinates must be recorded individually. In the context of our experiment, the analysis yields a set of 6 x- and y-coordinates for each participant. Because we are mapping the perceptual space using d-primes into an abstract Cartesian space, we have coined the term perceptual units (p.u.) as the unit of measure for both distance and area within this space. We refer to p.u. throughout, but as with any input dissimilarity matrix, the unit of measure will change in conjunction with the input units. We will also use the word *object* to refer to the perceptual category of the consonants used in our experiment. The distance between them or the area they occupy as a group refers to the computed perceptual distance or area between any two or more of these categories.

**Fig. 1:**
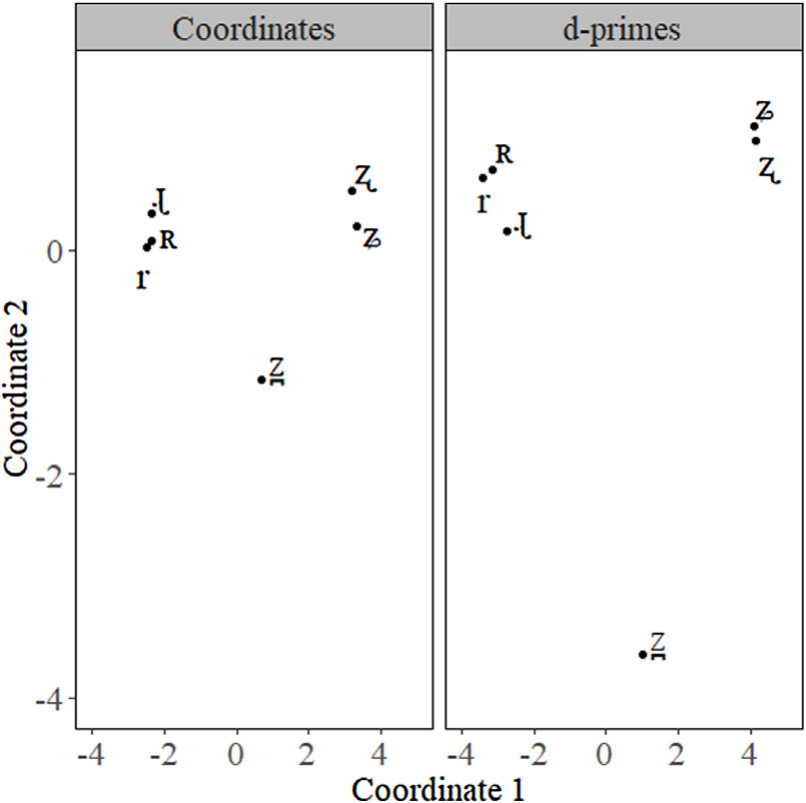
MDS solution involving our method of using the average coordinates of every participants MDS solution (left) versus the MDS solution involving the standard method of using the coordinates from the grand mean of the input dissimilarity matrix (right).

All the distance and area measures are performed using coordinates from each individual participant. Therefore, when plotting the results for visualization, the average of the coordinates is used for each object and not the average input dissimilarity matrix for all participants. In short, one of the main differences between our method and a more standard approach to MDS is that we have calculated the MDS solution for each individual participant and use the average coordinates of all participants as the basis for our MDS plot. The visual difference between the two methods can be striking. Fig. 1 presents the difference between the two strategies. On the left is our plotting method which involves calculating all the MDS coordinates for each participant and then calculating the average x- and y-coordinates for each object from MDS results. On the right is the standard method for plotting which involved calculating the average d-prime values for each comparison and then performing one MDS solution for the grand mean d-prime scores. The plot on the right will not accurately reflect the measurements made with our statistical method and ignores individual differences with the MDS solution. Note that both the distances and the relationship between the objects are different for the average coordinates and average d-prime solutions. Table 1 summarizes the different methods for each plot.

**Table 1:** Step by step differences between our method (coordinates) versus the standard method (average d-primes).

**Step**	Coordinates (our method)	Average d-primes (standard method)
1	Calculate an MDS solution for each of the participants using their respective d-primes as their dissimilarity matrix.	Calculate the average d-prime for each of the comparisons in the dissimilarity matrix.
2	Retrieve coordinates of each object in the MDS solution for each participant.	Calculate 1 MDS solution based on the average d-prime input dissimilarity matrix.
3	Calculate the average coordinates for each object.	Retrieve coordinates of each object in the MDS solution.
4	Plot the results.	Plot the results.

### Calculating and comparing distances between objects

**Fig. 2:**
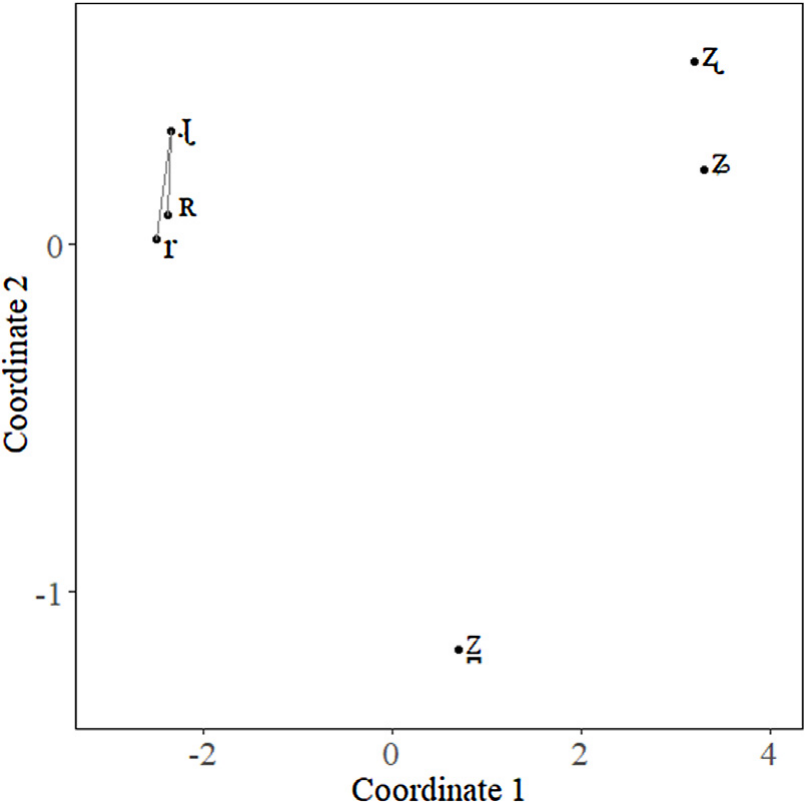
An example of the perceptual space we want to compare. Lines visualize the distances we are comparing between /r/ and /ɻ/ and /r/ and /ʀ/.

In an MDS solution, we can visualize the average coordinates of the objects once we have calculated the coordinates of each of the objects for each of the participants; however, in the traditional method, just because a pair objects appear further apart than another pair, we cannot be sure that they are statistically different from each other. Fig. 2 presents the MDS solution for the individual measures we have done. The lines between /r/ and /ɻ/, and /r/ and /ʀ/ represent the comparison we want to make. Visually, the distance between /r/ and /ɻ/ is greater than the distance between /r/ and /ʀ/; since the typical method of performing an MDS discounts variance in participants, we cannot be sure they are different. In the context of our experiment, the plot does not account for the fact that each participant has their own perceptual space. We have simply plotted the mean coordinates of each object within the perceptual space.

We can solve this problem by calculating distances between objects of interest. To do this we simply use a modified version of the Pythagorean Theorem presented in (1). In this formula, x_1_ and y_1_ represent the x- y-coordinates of the first object and x_2_ and y_2_ represent the x- y-coordinates of the second object.

(1) $$\text{Distance}=\sqrt{{\left(x_{2}-x_{1}\right)}^{2}+{\left(y_{2}-y_{1}\right)}^{2}}$$

**Fig. 3:**
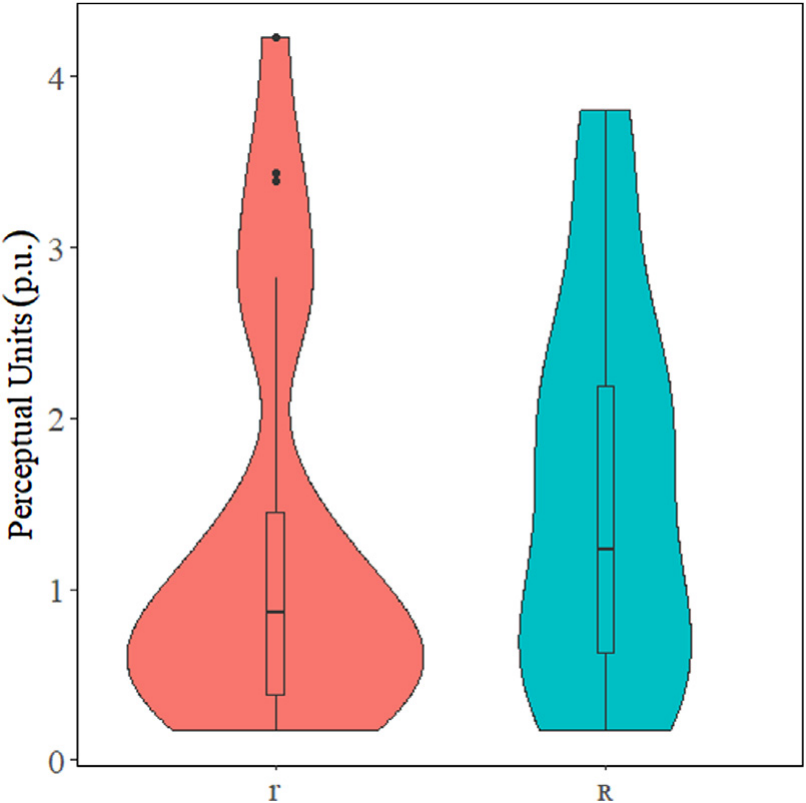
Violin plot for the distance between /r/ and /ɻ/ (left) and /ʀ/ and /ɻ/ (right).

The distance between the object of interest should be calculated for each participant. Following this, statistical tests can be applied to the results depending on the individual needs of the researcher. In our example, we used a simple Student’s *t*-test with the t.test() function in R [13] to compare the distance between each set of objects. Despite the visual suggestion that there was a difference between the two distances, the *t*-test reveals that the difference between the two was not significant [t(47.87) = −0.87, p = 0.389]. Fig. 3 presents a violin plot of the distances for each of the objects to /ɻ/.

### Calculating and comparing areas between groups of objects

**Fig. 4:**
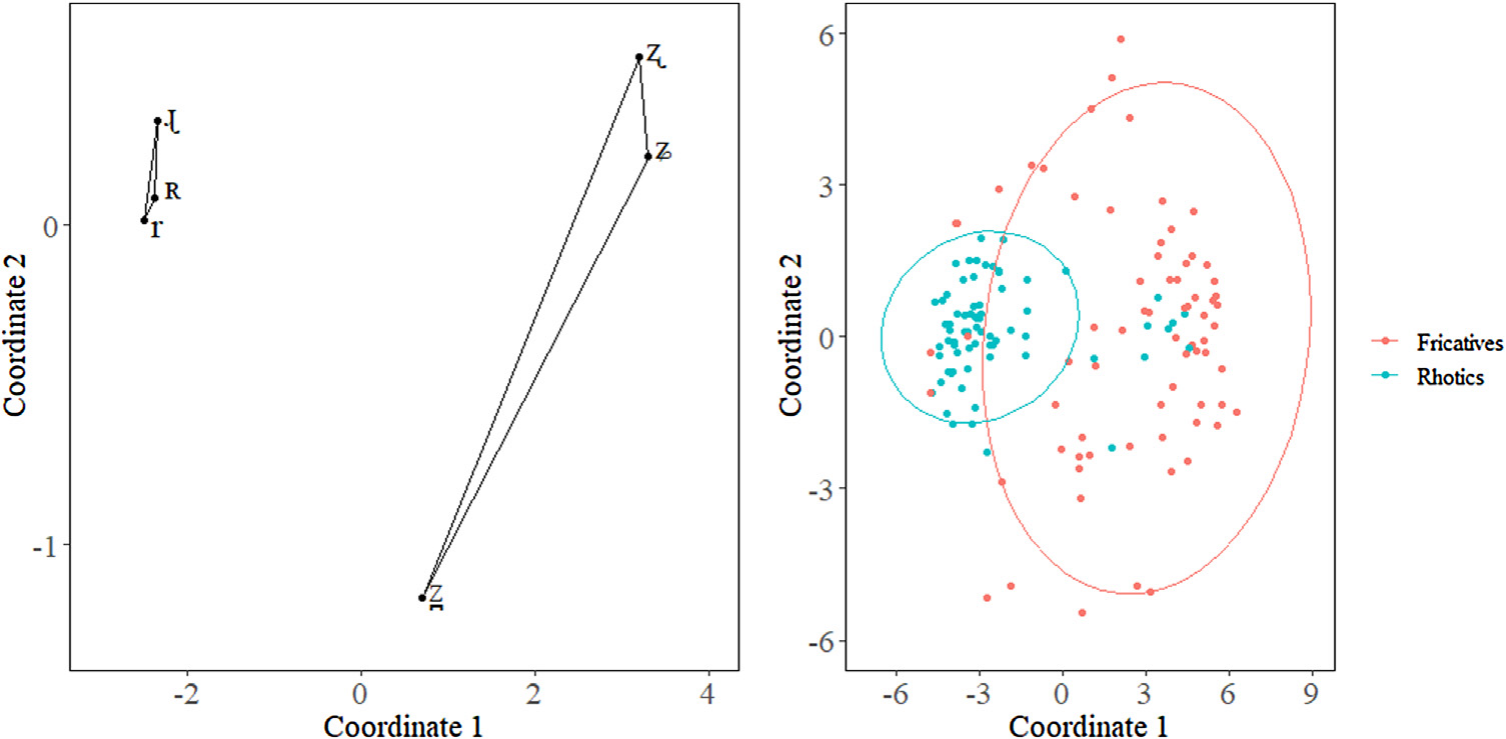
Plot of the rhotic and fricative groups we are comparing within the perceptual space (left); and a scatterplot with ellipses to show the variance in the groups (right).

It is also possible to compare how dispersed a group of objects are. In our dataset, we have two groups of objects in the perceptual space that we want to compare: rhotics, /r ɻ ʀ/, and fricatives, /z̪ ʐ ʑ/. Fig. 4 shows the groups we want to compare within the perceptual space. The rhotics are in blue and the fricatives are in red.

As before, we want to account for the variance due to each participant having a different perceptual space. Each participant’s perceptual space can vary on the relative position of each object and the relative distance between objects within the perceptual space. Our aim is to see if there is actually a difference in the dispersion of the rhotics versus the fricatives. Because both groups in our dataset contains 3 objects, each of the groups will always form a triangle. The further apart segments are (i.e., the more dispersed they are), the larger the area they occupy. Thus, we are operationalizing the area of the triangle formed by each group of objects as dispersion. To calculate the area of a triangle, we simply take the x- and y- coordinates of each of the objects and use the Surveyor’s area formula [14], also known as the Shoelace formula [15], presented in (2).

(2) $$\text{Area}=|\frac{\begin{array}{ccc} \left(x_{1}y_{2}-y_{1}x_{2}\right)+ & \left(x_{2}y_{3}-y_{2}x_{3}\right)+ & \left(x_{3}y_{1}-y_{3}x_{1}\right) \end{array}}{2}|$$

As with the previous example, x_1_ and y_1_ refers to the x- y-coordinates of the first object and so on. It does not matter which objects are 1, 2, or 3 for this formula and the area of any type of triangle can be calculated with this formula. The absolute value is used so that the area is always returned as a positive value. One limitation of this method is that if all the segments form a line, they will be in a collinear relationship and the area function will return as 0 irrespective of how dispersed the segments are.

Once the areas are calculated, it is again possible to use any inferential statistical measure. Again, we compared the areas using a Student’s *t*-test. The results reveal a significant difference in the dispersion of the two groups [t(24.93) = −72, p < 0.001]. The use of statistical comparison between the two groups adds evidence that there is an actual difference in dispersion.

It is also possible to calculate areas for groups of objects larger than three. This simply requires extending the Shoelace formula [15] to the corresponding number of objects in the polygon. The area of a polygon with any number of sides can be computed with the formula presented in (3).

(3) $$\text{Area}=|\frac{\begin{array}{cccc} \left(x_{1}y_{2}-y_{1}x_{2}\right)+ & \left(x_{2}y_{3}-y_{2}x_{3}\right) & \ldots & \left(x_{n}y_{1}-y_{n}x_{1}\right) \end{array}}{2}|$$

**Fig. 5:**
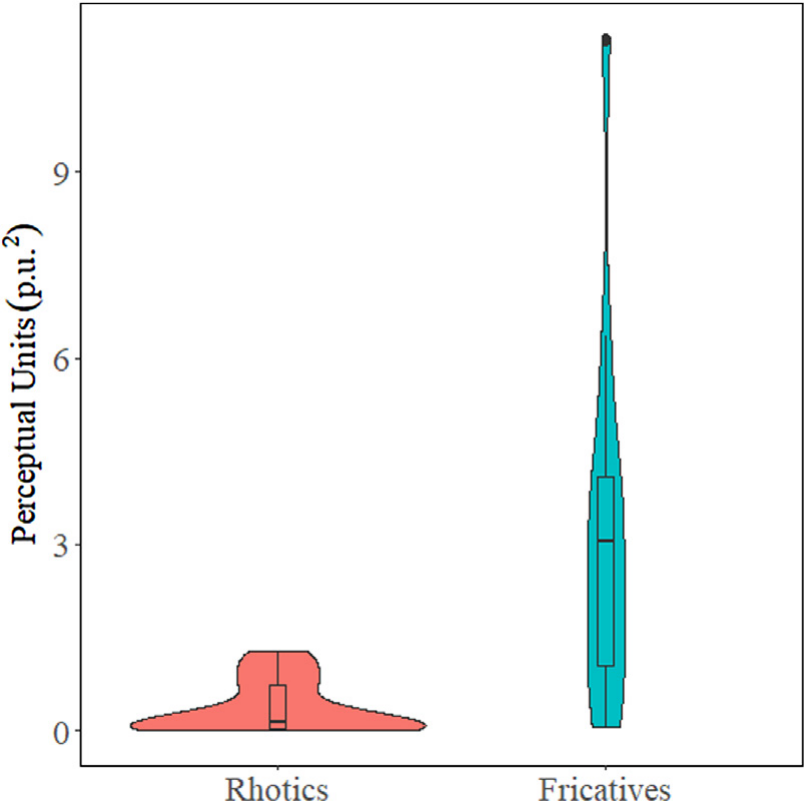
Violin plot of the area for the two groups, rhotics and fricatives.

**Fig. 6:**
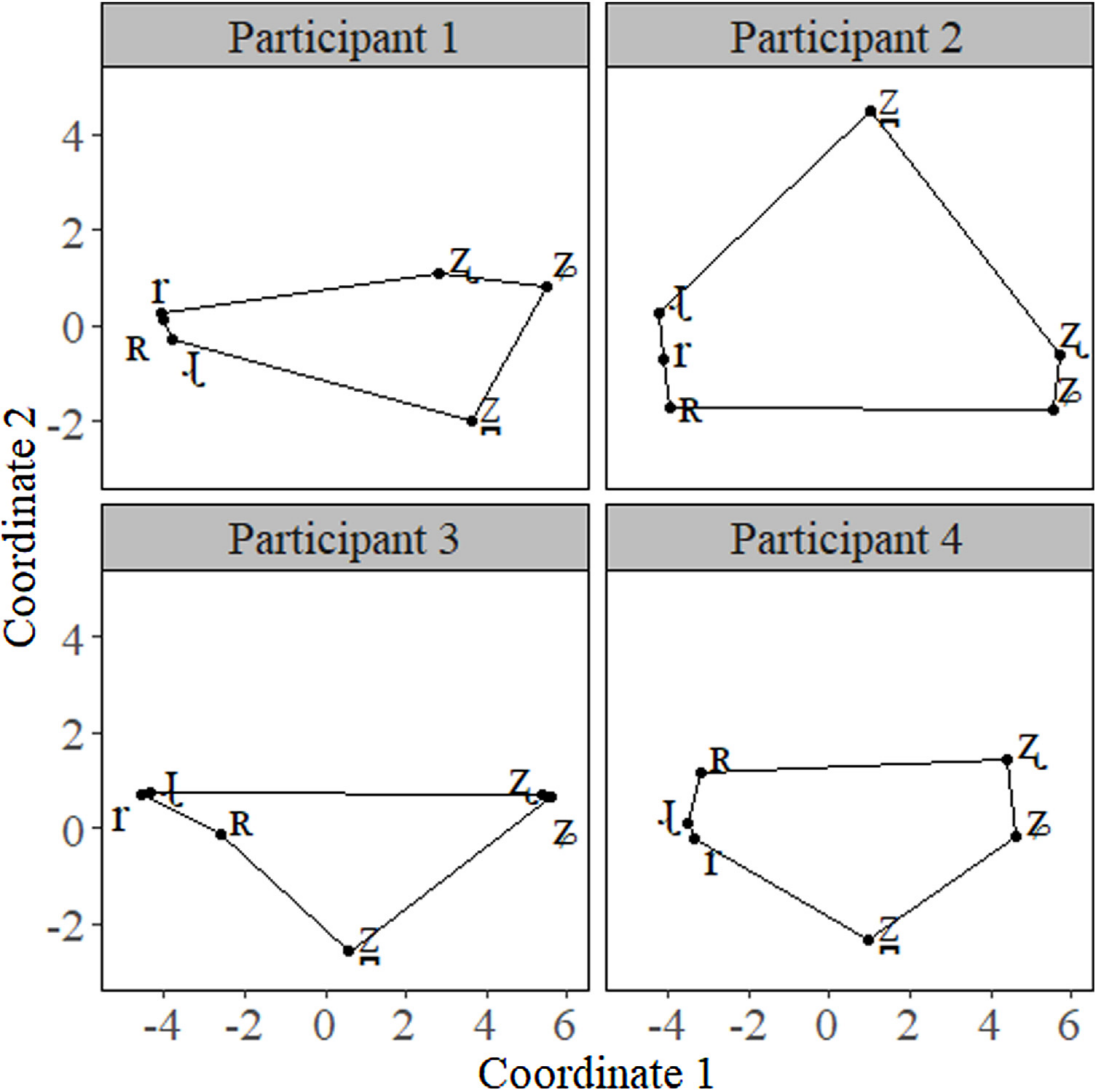
MDS solution for participants 1–4 in our perceptual experiment.

To execute this area calculation, we began with the x- y-coordinates of any vertex and proceed either clockwise or counterclockwise around the polygon until we reached the coordinates for the last vertex, x_n_ and y_n_. It is also important to note that this formula requires that the polygon produced by the x- y-coordinates does not form a self-intersecting polygon. In other words, none of the lines forming the edges of the polygon can cross each other. This is easy to avoid, but it requires that an MDS solution is plotted for each participant to observe the appropriate order to put each of the coordinates in for the equations. Fig. 5 presents the MDS solutions for participants 1–4 in our perceptual experiment. Note how the coordinates of each of the objects and the relationship between them are different for each participant. This is the reason we need to observe the distribution of each participant before calculating an area function of groups larger than 3 (Fig. 6).

Here, we present an example of calculating the areas of the entire perceptual space using the above formula and how that changes depending on the participant we are looking at. For ease, we will start each calculation with the x- y-coordinates of /ʐ/ and proceeding clockwise, although any vertex could be used for any of the calculations. It is important to remember that the polygon cannot have any intersecting sides, so the order for each of the participants is as follows: (1) /ʐ ʑ z̪ ɻ ʀ r/; (2) /ʐ ʑ ʀ r ɻ z̪/; (3) /ʐ ʑ z̪ ʀ r ɻ/; (4) /ʐ ʑ z̪ r ɻ ʀ/. If we follow the order set out for each of the participants, we arrive at the following solutions presented in Table 2 below. As with the other examples, the area for larger groups of objects can then be compared using the statistical method that suits the researcher’s needs.

**Table 2:** Example calculations for each of the participants 1–4.

Participant	Order	Solution (p.u.^2^)
1	/ʐ ʑ z̪ ɻ ʀ r/	16.95
2	/ʐ ʑ ʀ r ɻ z̪/	38.43
3	/ʐ ʑ z̪ ʀ r ɻ/	16.05
4	/ʐ ʑ z̪ r ɻ ʀ/	20.47

### Summary of our method

Multidimensional scaling is a powerful tool used in psychophysics and perceptual research, among other fields; however, since its inception, there have been issues in accounting for variance across individual participants because of the general use of averages across all participants for the dissimilarity matrix. Previous researchers have suggested using individual MDS solutions to find general trends or patterns [16], but they have been limited in their usability because of the necessity of large datasets. Our method generates a set of coordinates for each individual participant which can be used for inferential statistics, increasing the power of the conclusions that can be made with an MDS analysis. This method is particularly useful in determining if there is a difference in two or more distances between objects or if the area occupied between two or more groups of objects is different. This provides a useful tool for asking theoretical questions about human perception.

## References

[1] Gower J.C. (1966). Some distance properties of latent root and vector methods used in multivariate analysis. Biometrika.

[2] Gower J.C., Legendre P. (1986). Metric and Euclidean properties of dissimilarity coefficients. J. Classif..

[3] Cox T.F., Cox M.A.A. (2001). Multidimensional Scaling.

[4] Mead A. (1992). Review of the development of multidimensional scaling methods. J. R. Stat. Soc. Ser. D.

[5] Macmillan N., Creelman C. (1991). Detection Theory: A User’s Guide.

[6] Soli S.D., Arabie P. (1979). Auditory versus phonetic accounts of observed confusions between consonant phonemes. J. Acoust. Soc. Am..

[7] Shepard R.N. (1980). Multidimensional scaling, tree-fitting, and clustering. Science.

[8] Padgett J., Żygis M. (2007). The evolution of sibilants in Polish and Russian. J. Slav. Linguist..

[9] Johnson K. (2008). Quantitative Methods in Linguistics.

[10] Howson P.J., Monahan P.J. (2019). Perceptual motivation for rhotics as a class. Speech Commun..

[11] Creelman C.D., Macmillan N.A. (1979). Auditory phase and frequency discrimination: a comparison of nine procedures. J. Exp. Psychol. Hum. Percept. Perform..

[12] Ratcliff R. (1993). Methods for dealing with reaction time outliers. Psychol. Bull..

[13] R Development Core Team (2017). R: A Language and Environment for Statistical Computing. http://www.R-project.org/.

[14] Braden B. (1986). The surveyor’s area formula. Coll. Math. J..

[15] Meister A.L.F. (1769). Generalia de genesi figurarum planarum et inde pendentibus earum affectionibus. Novi Commentarii Societatis Regiae Scientiarum Gottingensis I.

[16] Ramsay J.O. (1969). Some statistical considerations in multidimensional scaling. Psychometrika.

